# Larger size and older age confer competitive advantage: dominance hierarchy within European vulture guild

**DOI:** 10.1038/s41598-020-59387-4

**Published:** 2020-02-12

**Authors:** Rubén Moreno-Opo, Ana Trujillano, Antoni Margalida

**Affiliations:** 1 Evolution and Conservation Biology Research Group, University Complutense of Madrid, E-28049 Madrid, Spain; 2 General Directorate on Biodiversity, Ministry for the Ecological Transition, E-28071 Madrid, Spain; 3 Bearded Vulture Study and Protection Group, Apdo. 45, E-25520 El Pont de Suert, Spain; 4 Institute for Game and Wildlife Research, IREC (CSIC-UCLM-JCCM), E-13005 Ciudad Real, Spain; 5 Division of Conservation Biology. Institute of Ecology and Evolution, University of Bern, CH-3012 Bern, Switzerland

**Keywords:** Behavioural ecology, Community ecology, Ecology, Zoology, Animal behaviour

## Abstract

Competition for limiting natural resources generates complex networks of relationships between individuals, both at the intra- and interspecific levels, establishing hierarchical scenarios among different population groups. Within obligate scavengers, and especially in vultures, the coevolutionary mechanisms operating during carrion exploitation are highly specialized and determined in part by agonistic behavior resulting in intra-guild hierarchies. This paper revisits the behavioral and hierarchical organization within the guild of European vultures, on the basis of their agonistic activities during carrion exploitation. We used a dataset distilled from high-quality videorecordings of competitive interactions among the four European vulture species during feeding events. We found a despotic dominance gradient from the larger species to smaller ones, and from the adults to subadults and juveniles, following an age and body size-based linear pattern. The four studied species, and to some extent age classes, show despotic dominance and organization of their guild exerting differential selection to different parts of the carrion. The abundance of these parts could ultimately condition the level of agonistic interactions. We discuss the behavioral organization and the relationship of hierarchies according to the feeding behavior and prey selection, by comparing with other scavenger guilds.

## Introduction

Competition for naturally limited resources generates complex networks of relationships between individuals, based on evolutionary, morphological and behavioral adaptations of different species and populations^1,2^. These competitive relationships at the intra- and interspecific levels may result in: (1) the establishment of dominance hierarchies between competing groups disputing a particular resource^3,4^; (2) resource partitioning, both in the means of obtaining the disputed resource and in resource selection^5^; and (3) differing energetic efficiencies obtained from different parts of a shared resource^6,7^.

In obligate scavenging animals, the coevolutionary behavioral mechanisms established within this guild are highly specialized in both competitive and facilitatory aspects^4,8–11^. This particular specialization in feeding demeanor is a result of the type of resource being exploited. Carrion is a pulsed food source which is unpredictable and usually limited in occurrence in space and time, which offers highly nutritive biomass^12–15^. In addition to ecomorphological adaptations (i.e. robust beaks, head without long feathers, accurate visual ability, digestive tracts tolerant to potential pathogens in carrion), obligate scavengers have developed behavioral adaptations to optimize carrion exploitation^16–18^, including various agonistic behaviors. Individuals exhibit aggressive activities to exclude competitors (con- or heterospecific) from disputed resources^19^. Such aggressive behaviors are common in many animal species^20^, although they are especially vigorous in terms of display and duration in predators and scavengers^21–23^. These groups do not need to devote much time to keeping watch for potential predatory attacks while feeding but must prevent competing scavengers from gaining access to the resource^24,25^. Depending on the relative success rates resulting from their agonistic behavior, dominance patterns or hierarchies can be established between different sympatric population groups^3^.

The agonistic behavior observed during carrion exploitation and the resulting intraguild hierarchies in obligate scavengers, especially vultures, has been the subject of several studies^24,26–30^. These studies have mainly concerned New World vultures and some African species, while in Eurasia this topic has been published from a descriptive perspective^24,31,32^ or for single species^33^. In contrast, studies of European vulture behavior during scavenging in relation to diversification, coexistence and specialization have been more detailed and extensive^18,23,34–37^. Information regarding the competitive interactions and dominance between European vultures helps us to understand the variety and complementarity of strategies present for the effective exploitation of the same resource by different species and age classes. Dominance relationships are relevant not only at the behavioral level, but also in community ecology, conservation biology, in addition to being an important determinant of the individual fitness^38^. It is also relevant to identify the relationships between the competitive ability of the different groups and their population numbers, or the group sizes gathering at carcasses, as well as to examine species´ morphological traits. In this sense, bill shape and size are features important in resource partitioning in vulture guilds^16,39^. Several studies have shown that indiviuals with larger body-sizes have higher hierarchical status^23,28,40,41^. As a result, dominance is probably determined by a combination of body size, prior ownership of carcasses, satiation levels, daily food intake expectations and the costs and benefits of agonistic encounters with conspecifics and heterospecifics^29^.

This paper revisits the behavioral and hierarchical organization within the previously poorly studied obligate avian scavenger guild comprising European vulture species jointly, based on their agonistic activities during carrion exploitation. We set out to identify patterns of individual dominance by identifying the types of competitive interactions that occur most frequently among European vultures, and whether the different species and age groups observed differ in their aggressive interactions. First, we evaluated the existence of intra- and interspecific competitive asymmetries in the four species of European vulture during scavenging activities, according to their success rates in agonistic confrontations. The initial hypothesis was that larger species with more powerful body parts (i.e., stronger beaks, greater claws, longer wings) have a higher hierarchical status and *a priori* win in a greater proportion of aggressive encounters. Second, in relation to age class, we hypothesized that adult birds would be competitively dominant in disputes over carrion compared with subadults and juveniles, due to their greater experience. Third, we tested whether the species and their different age classes differed in their roles as aggressor or the one receiving the aggression in any agonistic interaction. Here, we assumed that the larger species, those more frequently seen at the carrion, and the more experienced, older age classes would be more aggressive and dominant in obtaining and monopolizing food.

## Methods

### Study area and species

The study was carried out at four supplementary feeding sites (hereafter SFS) in southwestern Europe, two in the Pyrenean Mountain range (northeastern Spain, 42°25′41′′N 1°2′54′′E) and two in the Mediterranean Basin (central-western Spain, 39°10′55′′N 5°3′19′′W). The two regions were chosen to include representative populations of the four vulture species present in Europe^36^: the bearded vulture *Gypaetus barbatus* (BV) which is widely distributed across the Pyrenees; the cinereous vulture *Aegypius monachus* (CV) which is only common in central-western Spain; and the griffon *Gyps fulvus* (GV) and the Egyptian vulture *Neophron percnopterus* (EV) which are widespread throughout the two selected study areas^42^.

These species, which form the guild of European vultures, are all obligate carrion feeders and are physically adaptated to this food resource. They show different foraging strategies and carrion selection. The GV is the most gregarious species, attending in great numbers any feeding opportunity; this species prefers large amounts of carrion and entire carcasses of ruminants, mostly selecting soft tissues. The CV is the next most gregarious species and shows high numbers with small/medium-sized carcasses (rabbits or sheeps) or pieces of carrion, preferring tougher parts like muscles and tendons. The BV is a specialized bone-eater, mainly selecting extremities of different medium-sized ungulates. Juvenile and subadult individuals tend to concentrate around predictable sources of food, like supplementary feeding sites. Finally, adult EVs are non-gregarious visitors of both predictable and sporadic sources of carrion, mainly feeding on small peripheral soft tissues (skin, viscera, etc.)^18,34,36^. These species have differing population sizes, ecomorphological traits and territorial and migratory behaviors, which partly determine their conservation status and patterns of ocurrence while scavenging^43^. The GV is the most abundant species in Spain with more than 30,000 breeding pairs^44^, and overall consumes the largest amount of available biomass at feeding sites compared to other species^34,36^. In contrast, the CV and the EV are considered vulnerable, whilst the BV is endangered^45^.

### Study design and variables

We provided carrion on a monthly basis at each SFS on 100 occasions during two complete annual cycles from May 2009 to April 2011. Carrion was deposited during the day (randomly from 7:00 to 19:15 h GMT + 1) alternating different prey species (sheep *Ovis aries*, goat *Capra hircus*, pork *Sus scrofa*, cow *Bos taurus*, red deer *Cervus elaphus* and wild boar *Sus scrofa*), biomass (in kg) and distribution pattern (clumped and evenly spread) among the different SFSs [see details in 18,36]. Birds visiting the SFSs were monitored from the time of food provision until three days later using a high-resolution video camera (Arecont Vision Megavideo AV5100) connected to a computer, hard disk drive and rechargeable lithium batteries. The camera was located 20 m from the point of supply, allowing the observation of a wide area around the carrion (the 120° display angle of the camera also allowed the subsequent zooming of the recorded pictures with a sufficient sharpness to record species and behavior -see further details in^37^). The recordings of each feeding event were later visualized using the AV program v.5.1.4.239 Application Manager (Arecont Vision, Glendale, California, USA) to register the activity of individuals of each vulture species. Video analysis was performed by the same person to avoid observer biases in the data interpretation.

We selected one individual corresponding to each species and age class attending the SFS for each feeding event so the total number of individuals studied per feeding input varied (mean 4.15 ± 2.70 birds studied/feeding event). We aimed to homogenize individual observations and ensure that the circumstances that led to competition for the resource were even throughout our analyses, so we established as a condition that at least 10 individuals of two different vulture species were present at the time of selecting the vulture species under study. Similarly, all observations of behaviors overlapped with sufficient presence of carrion, estimated at least 25% of the amount biomass initially provided. For the less abundant BV and EV we randomly selected the birds appearing at the SFS. For the most gregarious species –GV and CV-, as their numbers were usually rather great at feeding events, we chose birds exhibiting distinctive plumage or non-feathered parts as well as those marked with plastic rings or wing tags for easy recognition and to avoid losing sight of them in the crowd during their entire period of stay. This resulted in behavioral records of 415 individuals (99 BV, 93 CV, 214 GV and nine EV, see Table 1).

**Table 1:** Summary of the number (n) and/or percentages (%) of the interactions observed related to agonistic behavior, and the interactions among the different species and age classes of European vultures during carrion exploitation.

	Bearded vulture				Cinereous vulture			Griffon vulture				Egyptian vulture
	*Ad*	*Sub*	*Juv*	*Tot*	*Ad* + *Sub*	*Juv*	*Tot*	*Ad*	*Sub*	*Juv*	*Tot*	Ad*
Observations of individuals (n)	34	36	29	99	75	18	93	72	74	68	214	9
Observations with interactions (n)	12	23	17	52	66	14	80	41	44	43	128	6
Observations without interactions (n)	22	13	12	47	9	4	13	31	30	25	86	3
Total interactions observed (n)	29	47	38	114	385	81	466	141	136	177	454	15
Mean interactions/observation	0,85	1,31	1,31	1,15	5,13	4,50	4,82	1,96	1,84	2,60	2,13	2,19
**Type of interactions**												
Fights/attacks (n)	12	21	23	56	247	65	312	50	63	137	250	2
% fights/attacks	41,3	44,6	60,5	49,1	62,5	81,2	65,6	48,5	46,6	64,6	55,5	13,3
Displacements (n)	17	22	14	53	137	14	151	42	62	62	166	11
% displacements	58,6	46,8	36,8	46,4	34,6	17,5	31,7	40,7	45,9	29,2	36,8	73,3
Stealing (n)	0	4	1	5	11	1	12	11	10	13	34	2
**Result of interactions**												
Success (n)	16	25	26	67	285	57	342	72	82	128	282	5
% success (success/interactions)	55,1	53,1	68,4	58,7	75,4	69,5	74,3	70,5	60,7	60,9	63,0	33,3
Failure (n)	9	18	9	36	71	22	93	10	36	71	117	8
% failures (failures/interactions)	31,0	38,3	23,6	32,7	18,7	26,8	20,2	9,8	26,6	33,8	26,1	53,3
Not clear, indiference (n)	4	4	3	11	22	3	25	20	17	11	48	2
Others -not clear, indiference- (%)	13,7	8,5	7,8	8,5	5,8	3,6	5,4	19,6	12,5	5,2	10,7	13,3
**Role in the interaction**												
Agressor (%)	26,3	43,7	37,9	36,0	74,0	63,0	68,5	56,3	49,5	52,3	52,7	8,3
Victim (%)	73,7	56,3	62,1	64,0	26,0	37,0	31,5	43,6	50,4	47,6	47,2	91,6

The number of observations of the different species and age classes are shown, categorized into the competitive interactions recorded, as well as the global numbers for each of the categories of the response variables considered in the analyses (*Type of interactions*, *Result of the interactions* and *Role in the interactions*). Age classes: Ad (Adults), Sub (Subadults), Juv (Juveniles) and Tot (Total = AD + SUB + JUV).

*We only detected adult Egyptian vultures during our observations of feeding behavior at feeding sites.

Data collection during the visualization of the video images began with the random selection of an individual. Its activity was then recorded from the time of its appearance until it disappeared from the visible recording area (this constitutes an *observation* in the data presented here). The duration of the continuous presence of the selected vulture in the recording area around the carrion averaged 22 minutes and 3 seconds ± 41 minutes and 43 seconds (minimum 4 seconds; maximum 4 hours, 26 minutes and 25 seconds). We first noted if the targeted individual interacted with another individual, to evaluate the interaction rates for each observation. For the purposes of our study, we considered an *interaction* as the direct action of a vulture to another vulture in which aggressive activity resulted from competition for food or another associated activity, such as the occupation of a certain place in the vicinity of the carrion (see Table 2). For each *individual observed*, we recorded its interaction with another individual, referred to here as the *individual interacted*, identifying their species and age classes and assigning the interaction to the categories of response variables indicated below (Table 2). The interaction was considered complete once both individuals physically separated and the aggressive activity ceased. Each individual observed might perform several interactions during the same observation, 39 being the maximum recorded. We also recorded the interactions exhibited by other vulture/s directed towards the *individual observed*, to reveal their role as perpetrators or victims of the interaction.

**Table 2:** Description of the variables used in the study of agonistic behavior and interactions among European vultures during carrion exploitation in Spain including the different categories into which these variables were divided.

Variable	Categories	Description
Age class*	Adult	Birds showing definitive adult plumage, according to^48^
	Juvenile	Birds with plumage traits corresponding to their first and second calendar year
	Subadult	Birds with plumage traits corresponding to their third and fourth calendar year (up to sixth calendar year in the bearded vulture)
*Type of agonistic interaction*	Fight/attack	Aggression or attempted aggression by using beak, claws and/or opening the wings to occupy the place occupied by another individual
	Displacement	Charge/push to move another individual and occupy its place, without the help of beak, claws or open wings
	Stealing	Removal of a piece of food from an individual which had already acquired it, either from its beak or claws, or by forcing regurgitation
*Result of the interaction*	Success	The individual observed managed to displace the other individual from its place, or to steal food from it
	Failure	The individual observed failed to displace the other individual from its place, or failed to steal food from it by being repelled or avoided
	Not clear/indifference	No change of place or stealing from one individual by another (aggressor or victim) or the result is not clear
*Role in the interaction*	Aggressor	Individual starting and carrying out competitive interactions on another individual
	Victim	Individual initially receiving and suffering the interaction (fight/attack, displacement, stealing)

All of the variables were considered as response variables except *age class*, which was explanatory.

*For the cinereous vulture we joined the adult and subadult age classes in the analyses due to the variability in plumage traits after the juvenile phase^48^.

Several other variables were sampled during the observational monitoring of each vulture on the basis of their linkage to agonistic behavior to, and dominance over, other individuals as well as to the competitive interactions observed^30,39^. The following response variables were included in the statistical analyses: *Type of interaction, Result of the interaction* and *Role in the interaction* (Table 2), following the categories and information used in previous studies^29,46,47^. We analyzed five explanatory covariates: *species observed* for the randomly selected individual, the *age class of the individual observed*, the *species interacted* (the individuals interacted by or to the observed vulture), the *age of the individual interacted* (Table 2), and the *biomass* in kg supplied as carrion during each feeding event. We included this latter variable as a proxy of the size and availability of food, which in fact could modulate the type and results of interactions^18,23,35^.

The corresponding physical traits of the birds for denoting their age class and the assignment of the different behaviors were confirmed by zooming into the high-resolution images (at 5 Mpixels resolution). The individuals of the target species could not be weighed nor sexed due to the lack of observable sexual dimorphism in these species^48^. We consequently considered the body mass for each species following^49^ and taking the lower value of the range for further analyses (7.0 kg for CV, 6.0 kg for GV, 4.5 kg for BV and 1.6 kg for EV, Table 3).

### Statistical analyses

First, we compiled a table with the totals and percentages obtained for each of the variables analysed (Table 1).The database with all the observed interactions was analysed to determine the differences in agonistic behaviors between the various species and age groups. Aiming at recognizing what type of explanatory variables, and their interactions, influenced to a greater extent aspects of hierarchical dominance, we created three Generalized Linear Models (GLZ), one for each of the three categorical response variables considered (Table 2). We performed a Type 1 Likelihood Ratio (LR) test to find the log-likelihood value, the Chi-square statistic (χ^2^) and the significance value (p) for each of the explanatory variables evaluated as well as for their interactions. All of the models developed included a logit function; the *Type of interaction* and *Result of the interaction* response variables showed a multinomial distribution of errors and the variable *Role in the interaction* had a binomial distribution. Because our models were based on categorical data and the limitations derived from our design were too large to compute reliable estimates, only the following three categorical explanatory covariates could be included in the models: *species observed*, *age class of the individual observed* and *species interacted*, together with the continuous covariate *biomass*. The models reported which of the explanatory variables, as well as the interactions between them, showed a greater likelihood that the observed data is most probable (log-likelihood) as well as which had a more robust level of significance (p). However, because the variable *age of the individual interacted* can influence the outcomes of paired interactions, individualized χ^2^ analyses were performed for each of the three response variables related to *age of the individual interacted*.

We finally performed a General Regression Model (GRM) analysis to check the influence of body size and age on percentage success in the interactions recorded with the purpose of obtaining a global pattern between dominance rates and the size and the age of the individuals involved. For that, we considered the results of percentages of successful interactions of each group of individuals (Table 1) as a continuous response variable and the body mass and age class of the considered group as explanatory variables. The statistical analyses were conducted using *Statistica* 6.1 (StatSoft, Tulsa), and a standard p value of 0.05 applied.

## Results

We recorded agonistic interactions with other individuals in 64.1% of observations of individual vultures (n = 415) taken from >7,500 hours of visualized video recordings (Table 1). The total number of agonistic interactions registered were 1,049. The mean number of interactions in each observation was 2.5 ± 1.5; CV being the species with the highest number of interactions towards other species (4.8 interactions/observation), followed by GV, EV and BV (Table 3). The majority of the interactions (65.0%) were intraspecific and the rest were between individuals of different species.

**Table 3:** Ranks of the different vulture species and age classes based on the percentage of successful interactions, and body mass (in kg^49^,) and age class^48^.

Species	Rank (%)	Body mass (kg)
Cinereous vulture	1 (74.3)	7.0–12.5
Griffon vulture	2 (60.9)	6.0–10.0
Bearded vulture	3 (58.7)	4.5–7.1
Egyptian vulture	4 (33.3)	1.6–2.4
**Species**	**Age class**	**Rank (%)**
Cinereous vulture	Adult + subadult	1 (75.4)
Griffon vulture	Adult	2 (70.6)
Cinereous vulture	Juvenile	3 (69.5)
Bearded vulture	Juvenile	4 (68.4)
Griffon vulture	Juvenile	5 (60.9)
Griffon vulture	Subadult	6 (60.7)
Bearded vulture	Adult	7 (55.1)
Bearded vulture	Subadult	8 (53.2)
Egyptian vulture	Adult	9 (33.3)

**Figure 1:**
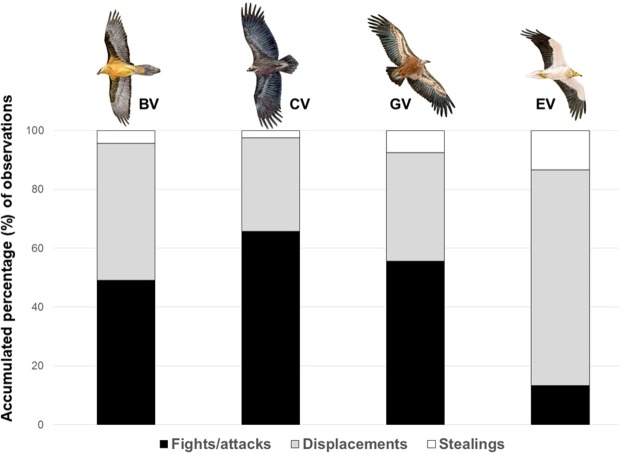
Accumulated percentage of the number of observations recorded for each of the three types of interactions (*fights/attacks*, *displacements* and *stealing*) related to agonistic behavior in the four European vulture species (BV = bearded vulture *Gypaetus barbatus*, CV = cinereous vulture *Aegypius monachus*, GV = griffon vulture *Gyps fulvus*, EV = Egyptian vulture *Neophron percnopterus*). Pictures courtesy of J. Varela (http://www.juanvarela.com/).

**Figure 2:**
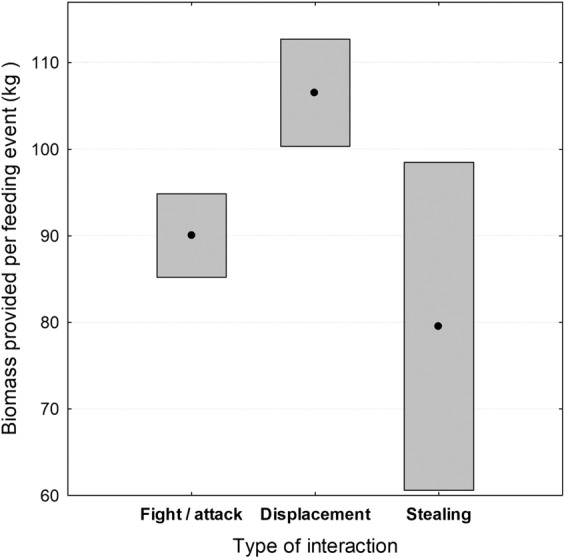
Mean (black dots) ± 95% confidence interval of the biomass (in kg.) supplied at each feeding event for which the different types of interactions were registered.

Regarding the type of agonistic interaction observed (see Table 2), the most frequent (58.8%) was *fights/attacks* against other birds. In contrast, food *stealing* was rarely observed (5.0%), and was mainly performed by GVs (Table 1). The type of interaction observed was best correlated with the *species observed* (Log-likelihood = −797.30; χ^2^_6_ = 29.38; p < 0.001), followed by the *age class of the individual observed* (Log-likelihood = −784.91; χ^2^_4_ = 24.77; p < 0.001) and the interaction between the species observed*species interacted (Log-likelihood = −767.07; χ^2^_8_ = 15.85; p = 0.044). The *age of the individual interacted* (χ^2^_2_ = 54.44; p < 0.001) as well as the *biomass* provided at each feeding event (Log-likelihood = −811.99; χ^2^_2_ = 19.63; p < 0.001) also modulated the type of interaction. The BV instigated approximately the same number of *fights/attacks* as *displacements*, whereas CVs and GVs performed a higher proportion of *fights/attacks*. Displacement was the most common type of interaction provoked by EVs (73.3%, Fig. 1). With respect to the different age-classes, in all of the species both adults and subadults showed a similar ratio between the types of interactions instigated (around 55% of fights/attacks and 40% of displacements). In contrast, juveniles were proportionally more aggressive towards other species, their percentage of fights/attacks being 68.2%. When acting as victims, non-adult individuals received a higher percentage of *fights/attacks* (84.8%) and *displacements* (64.6%) than the adults, and a similar ratio of *stealings* (51.1%). It is noteworthy that *stealings* always occurred at the intra-specific level in GVs (n = 34), BVs (n = 5) and EVs (n = 2), while in CVs (n = 12) one third of the attempted thefts were directed towards GVs and the rest to other CVs. The lesser amount of carrion supplied at each feeding event the more aggressive interactions registered (Fig. 2); when food was scarcer, activities like thefts and fights were more frequently observed.

**Figure 3:**
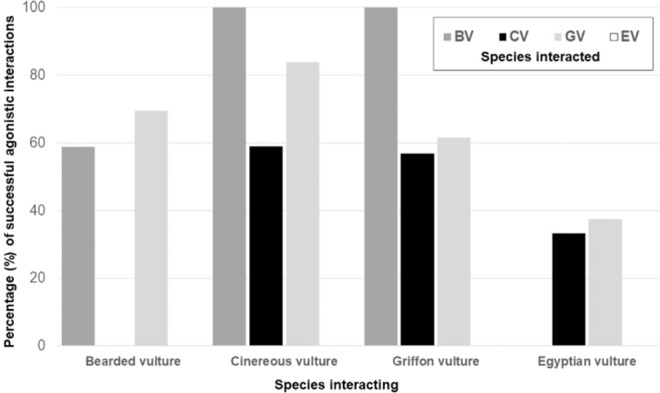
Percentages of successful agonistic interactions (i. e. the *observed individual* manages to displace or steal food from another individual) recorded in *observed individuals* for each of the four species of European vultures in relation to *interacted individual* of those same four species (BV = bearded vulture *Gypaetus barbatus*, CV = cinereous vulture *Aegypius monachus*, GV = griffon vulture *Gyps fulvus*, EV = Egyptian vulture *Neophron percnopterus*). The species of the *observed individuals* (and their percentage success) is shown on the x-axis, and the species of the *interacted individuals* is indicated by different coloured columns along the y-axis.

The results of competitive interactions (success, failure or not clear/indifference) were significantly dependant on the *species observed* (Log-likelihood = −768.64; χ^2^_6_ = 15.93; p = 0.014), the *age class of the individual observed* (Log-likelihood = −753.60; χ^2^_4_ = 30.06; p < 0.001), the *species interacted* (Log-likelihood = −743.86; χ^2^_6_ = 19.48; p = 0.003) and by the interactions between species observed* age class of the individual observed (Log-likelihood = −732.16; χ^2^_8_ = 23.40; p = 0.002) and species observed*species interacted (Log-likelihood = −722.56; χ^2^_10_ = 19.19; p = 0.037). The *biomass* provisioned modulated the result of the interaction (Log-likelihood = −776.60; χ^2^_2_ = 32.52; p < 0.001), such that when there was less food available the success rate in the interaction was higher. The *age of the individual interacted* also influenced the success ratio in the interactions (χ^2^_2_ = 14.68; p < 0.001). The highest success rates in agonistic interactions were generally achieved by adult and subadult CV (Table 3). The next highest were shown by adult GVs and juvenile CVs, while the lowest were shown by adult and subadult BVs and EVs, which therefore showed the least dominance (Table 3). The success/failure ratio also varied depending on the interacting species. The percentage of success in conspecific confrontations was similar for BVs, CVs and GVs (around 60%, Fig. 3); that is, when an individual of these species instigated an interaction. At the heterospecific level, CVs launching an interaction obtained high levels of success against BVs and GVs, while GVs and BVs only won more than 60% of the observations when competing with each other. The EVs observed were generally unsuccessful against all other species (Fig. 3). In relation to age class, adults obtained the highest rates of success, especially when facing juveniles (83.3%) and subadults (80.5%). In contrast, subadults and juveniles showed lower success rates, especially when competing with adults (38.4% and 45.8% respectively). The general success percentage decreased to 20.7% when the *age of the species interacted* was adult.

**Figure 4:**
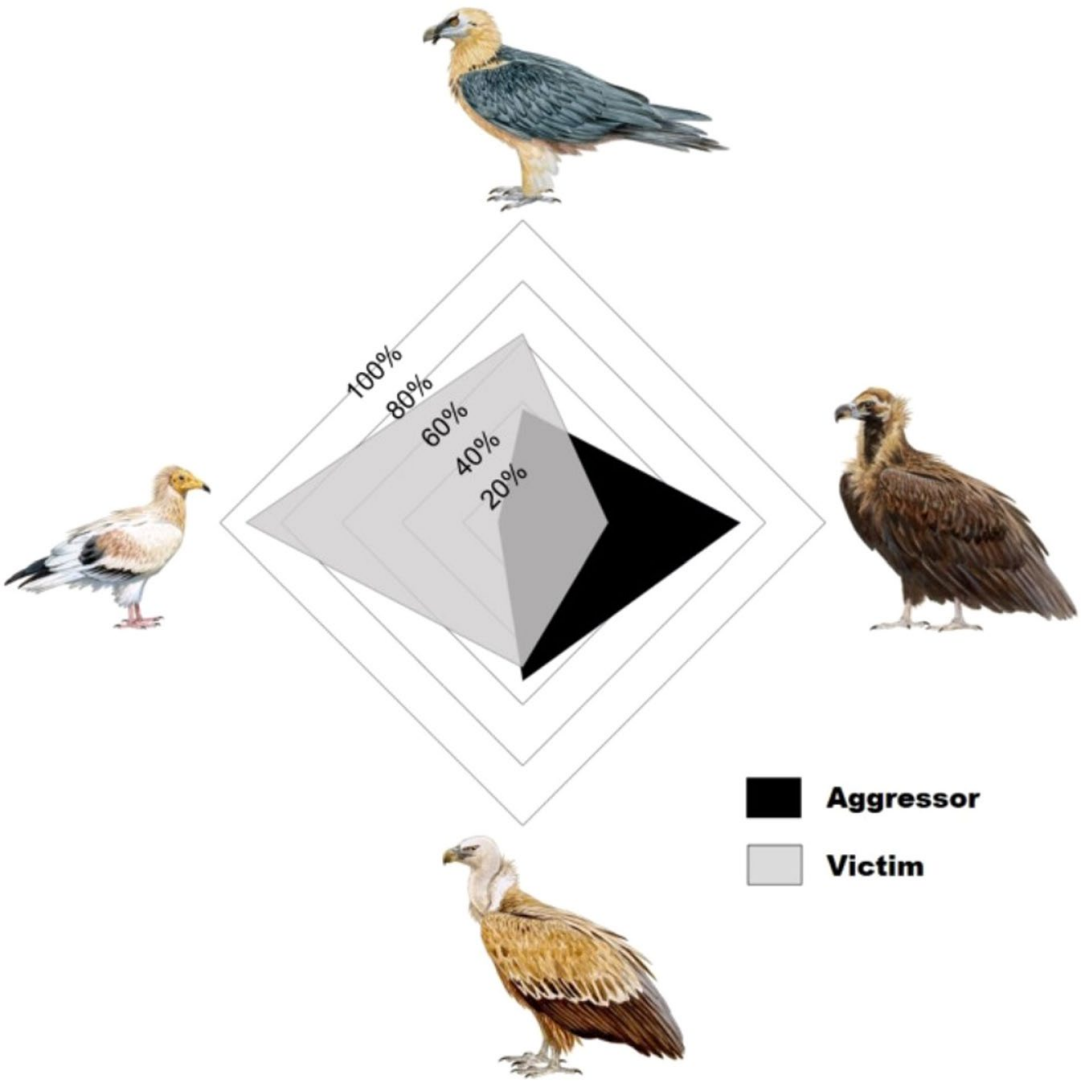
Role as aggressor or victim in the four European vulture species (above = bearded vulture *Gypaetus barbatus*, right = cinereous vulture *Aegypius monachus*, below = griffon vulture *Gyps fulvus*, left = Egyptian vulture *Neophron percnopterus*) based on the proportion of agonistic inter-specific interactions in which the individuals observed during carrion exploitation behaved as aggressor or victim. The outermost concentric percentage line indicates 100% and the innermost indicates 20%. Pictures courtesy of J. Varela (http://www.juanvarela.com/).

Only the *species observed* had a significant influence in relation to its role as an aggressor or victim in an aggressive interaction (Log-likelihood = −508.40, χ^2^_2_ = 72.39, p < 0.001). CVs were the most likely to initiate aggressive encounters (71.9% of their interactions were as aggressors) compared with the next highest GVs (52.3%), while EVs and BVs were most likely to be the subject of aggressive behaviors (91.6% of and 62.5% of their interactions, respectively, Fig. 4). In contrast, there were no significant differences between the different age classes in their role as aggressor or victim (Log-likelihood = −508.66, χ^2^_2_ = 1.63, p = 0.441).

The summatory model explaining the success rates of competitive interactions (Table 1) was positively related to increasing body mass and older age classes of vultures, as well as to the interaction of both variables (R^2^ = 0.98; p = 0.006; Table 4).

**Table 4:** Results of the General Regression Model (GRM) assessing the percentage of successful interactions induced by agonistic behavior in relation to the body mass (kg.) and age class (adult, subadult and juvenile) of each group of vultures studied during feeding activity.

Variable	Sum of squares	Degrees of freedom	F	p
Intercept	775.29	1	114.19	0.001
*Body mass*	67.77	1	9.98	0.050
*Age class*	209.11	2	15.40	0.026
Body mass*Age class	199.35	2	14.68	0.028
Error	20.36	3		

## Discussion

### Behavioral organization within the guild

In our study, the agonistic behaviors around carrion were grouped into three distinct types according to the level of aggression. The most frequent and aggressive were *fights/attacks* in which the vultures used their beaks, claws or opened wings to attack or try to attack, either to displace a competing vulture or to maintain their own position. In this type of interaction, physical contact was observed and serious, possibly even lethal, wounds might be inflicted^32^. Juvenile vultures of the studied species engaged in such fights more frequently both to con-and heterospecifics, possibly either because of their lack of experience and, consequent lower appreciation of the risk of fighting, or because their age class is more susceptible to starvation/dehydration^50^ and is prepared to take greater risks. *Displacements*, which comprised more than a third of the interactions observed, were less aggressive in nature and were more commonly performed by the less belligerent species. Finally, food *stealing* attempts were considered as an independent category of interaction since they concern kleptoparasitic behavior, common among obligate and facultative scavengers^51–53^. The rarity of food *stealing* from individuals of other species might indicate pronounced resource partitioning and differential carrion selection behavior between the four species^18,36^.

Our results showed that among the four European vulture species, the CV is dominant: they exhibited the highest agonistic behavior success rates, had a greater number of interactions per observation, were the most frequent aggressor and most often showed *fights/attacks* behaviors. CVs, especially the adults and subadults, have adaptations which make them especially effective against other species and age groups. Their greater body size and more powerful beaks and claws enable better competitive performance against the other vulture species. Likewise, they have a number of displays and postures not present in other species, e.g. bristling of the plumage and behaviors such as the “threatening march”^24^.

The GV is next on the hierarchical scale with the second greatest number of successful confrontations, especially in the case of adults^27^. The GV´s second rank in the interspecific hierarchy is probably also related to its body size and common behavior of scavenging at gatherings, so that individuals can gradually gain experience in the numerous struggles against other individuals to obtain limited food. The most commonly observed GV behaviors were fights to maintain a preferential position close to the carrion -where most biomass is available^23,27^- and to defend individual pieces of food from stealing by other GVs and CVs.

Regarding the BV, juvenile individuals were more competitive than adults and subadults, and even more successful than juvenile and subadult GVs, showing the bold and social nature of that age class^49^. Juvenile BVs are more common at supplementary feeding sites (SFS), with their predictable food supply, compared with the territorial adult population^36,54^, which could explain their greater number of interactions per observation and their effectiveness in clashes with other vultures, especially other subadult and juvenile BVs and juvenile GVs. Finally, the EV was confirmed as the most passive of the guild since most of its interactions resulted from the aggression of the other species, especially GVs. Its smaller relative size, lower number at SFS and its means of food acquisition (see below) may explain its low success rate in confrontations.

### Relationships between hierarchies and feeding behavior

A species´ feeding behavior and the degree to which it is used to dealing with other scavengers at crowded carrion sites could in part explain the frequency (mean number of interactions/observations), types and results of interactions found in the vultures in our study. First, we have verified how carrion availability determines both the types of interactions and their result. When there was less food, more aggressive interactions (thefts and fights) happened due to increased competition for limiting resources (Fig. 2), which was also influenced by the number of birds present^34–36^. In this sense, as our findings show, the species most commonly observed in this competition and that monopolize most of the resources (i.e. the GV^34,36^ and also the CV), would have a greater probability of success when resources are limited.

The CV frequently attends feeding events both at SFSs and naturally occurring carcasses, but is less frequently found in the scrums and clamours formed by numerous GVs around a carcass. On the contrary, CVs are more competitive in exploiting separated parts of a carcass when the number of GVs is lower^36^. It is also common for them to seize a small or medium sized isolated piece of carrion which they eat and defend^55^. CVs usually win agonistic encounters launched on other, preferably single, vultures in order to snatch small carcasses or pieces of muscle, skin or tendons^36^. This more aggressive and successful behavior would enable CVs to select and obtain choice carrion parts, which could be in limited supply in natural large cadavers (e.g. large herbivores) or in the piles of carrion provided at SFSs.

The GV is the most abundant vulture at scavenging events in Europe and takes the majority of the available carrion biomass overall^34,36^. It prefers the most abundant parts of carcasses such as the viscera and muscles, so large groups gather at large herbivore carcasses, favored by social facilitation (i.e. the processes in which conspecifics provide information about the position of food;^8,9,35^). Its high position in the hierarchy at feeding events allows it to occupy the most favourable positions around a carcass and so to have the best access to the soft parts of the animal^35,36^. It also competes with other species for the scattered pieces of muscle/tendon that are also sought by CVs, and CVs are at a disadvantage if the numbers of GVs around those parts are too numerous.

BVs are specialist bone feeders^18,56,57^ and >3/4 of their interactions observed in our study were directed towards conspecifics. Juveniles and subadults spend twice as much time as adults scavenging on the ground^18^ and showed greater agonistic activity with the purpose of obtaining the most appropriate size and shape bones such as ribs, vertebrae, legs and hooves^57^. In contrast, adults usually locate and select the most favourable bones while still in the air and then carry them to other places outside the crowd once they are taken^18^. This practice of swooping down to pick up a bone before flying off with it would certainly contribute to their low observed interaction rate and the heterospecific interactions that did occur were mainly due to attacks by GVs that had detected active BVs.

The EV is the smallest and least dominant of the European vultures and minimises its interactions with other species at feeding events by almost always taking up a position at the edge of the feeding crowd and consuming small pieces previously discarded by GVs and CVs^18,36,58^. EVs rarely provoked interactions but suffered aggression from heterospecifics trying to displace them to take pieces of scattered carrion. Despite these apparent competitive disadvantages at SFSs and other feeding points attended by many vultures, EVs positively select these events and locations^34,36^ because the resource availability is higher and more predictable, thus potentially improving their chances of survival^59,60^.

### Comparisons with other scavenger guilds

The establishment of hierarchies during carrion exploitation has also been shown in many other scavenger species. Regarding facultative carrion-eating birds, hierarchies in favour of larger species have been observed in some scavenger communities^61^. At the intraspecific level, older, larger males tend to be dominant, such as in the bald eagles *Haliaaetus leucocephalus*^50^ and carrion crows *Corvus corone*^62^, while in other cases such differences have not been observed (e.g. in the sea eagle *Haliaeetus albicilla*^63^).

Obligate scavengers show clear dominance hierarches and the behavioral patterns observed in our study of European vultures resemble those found in the vulture guilds of the Americas and Africa. Body size as a determining factor in the success rates of competitive interactions shows a similar pattern in American vulture guilds as in European ones; larger species are progressively dominant over smaller ones (i. e. Andean condor *Vultur gryphus* > king vulture *Sarcoramphus papa* > black *Coragyps atratus*/turkey vulture *Cathartes aura* > lesser vulture *Cathartes burrovianus*^28,39^). The same trend applies for African vultures (lappet-faced vulture *Torgos tracheliotus* > Rüppell´s vulture *Gyps ruepellii* > white-backed vulture *Gyps africanus* > white-headed vulture *Trigonoceps occipitalis* > hooded vulture *Necrosyrtes monachus* > Egyptian vulture *Neophron percnopterus*^10,26,64–66^). When considering body size within the same species the largest individuals also dominate smaller ones, as seen in the Andean condor and the turkey vulture^29,30^. We were unable to examine the effect of differing body size in conspecifics in our study due to the lack of visible differences in body size between the different age or sex classes of the species we studied^48^.

The patterns of a higher hierarchical position for adults over preadult age classes obtained in our study resembled those of New World vultures^28,30^. Moreover, sex was also a key factor in Andean condors^30^. However, the situation of European vultures could differ since, unlike Andean condors, they have a moderately reversed sexual dimorphism (i. e. females are slightly larger than males^43^), so females could increase their competitiveness against males following the global pattern of positive dominance related to body size^60^.

In general, we would expect the dominance ranks among the different vulture guilds to be similar. First, Old World species would form three Genus groups based on phylogenetic similarities: *Aegypius*/*Torgos*/*Trigonoceps/Sarcogyps* > *Gyps* > *Neophron*/*Necrosyrtes/Gypaetus*^67^. Each of the three proposed groups have similar morphological and behavioral traits during carrion exploitation so they occupy a similar hierarchical position in all the areas in which feeding behavior has been studied^16,24^. Similarly, the different European vulture species have trophic niches similar to those of New World species, both in diet selection and in their behavior during feeding: CVs are similar to Andean Condor, and GVs are similar to black and turkey vultures in this respect^30,39,68^.

### Implications on community ecology

The existence of a hierarchically structured organization in animal/plant guilds linked to a specific resource shows the complex framework of social relationships as well as competition and facilitation processes^1,69^. The study of vultures allows the direct observation of agonistic behaviors around a trophic niche, such as carrion, offering a great energy input and appearing scattered in both space and time naturally. But this is not the usual pattern of availability of food in the wild for other niches, which usually occurs at broader scales^70^, so that intra- and interspecific competition often have to be evaluated through indirect methods, rather than individual behaviors observed directly through video recordings.

Our results reinforce a common behavioral pattern also detected in other groups of animals competing for a limited food resource: more experienced individuals with better physical condition occupy areas of optimal availability and quality of resources while suboptimal patches are exploited by younger and less competitive individuals^62,71,72^. This triggers relevant effects on their fitness, especially in terms of survival and breeding output, favouring the older and larger individuals^73,74^. In passerines, this has been shown, for instance, at wintering locations where both migratory and sedentary birds of the same and different species are present so that local, more experienced and better body conditioned individuals are more abundant in areas with a greater abundance of fruits of temporary occurrence^75,76^. Moreover, predatory birds and mammals compete for the occupation of territories with better prey availability and quality^77,78^. Thus, they tend to exhibit aggressive behaviors to expel other potentially competing individuals through struggles and exhibitions that are more frequently won by experienced and larger individuals^79^. In general, this selection by territorial species towards the best feeding and, consequently, high quality breeding habitats generates better breeding performance. Although it can also provoke a reduction in territory size and a greater investment in vigilance and aggressive interactions which, sometimes, leads to density dependence phenomena affecting demographic parameters^80^.

In relation to the above, and according to our results, animal communities find a balance between agonistic behaviors and resource partitioning. Competition and aggression establish dominance structures at the intra- and interspecific levels but their subordinate effects are attenuated by a differential selection towards different parts of the resource allowing the exploitation of separated ecological niches^81–83^. This has been previously studied in nested communities and assemblages, both in different habitats and for taxonomically related guilds^84–86^. Consequently, adaptations for exploiting the same resource generate a dynamic balance in animal communities. Avian scavengers as a study model show the influence of both interrelated elements: agonistic behaviors for the dispute of a common resource cause dominance ranks that are modulated due to a differential selection of different parts of the resource so that most species and age groups can fullfil their ecological needs^18,85,87^.

## Conclusions

We provide the first detailed observational study of the relative agonistic behavior of the four European vulture species, which result in despotic dominance patterns during carrion exploitation. Our results describe their species specific behaviors based on quantified and standardized annotated criteria and the hierarchical classification between individuals of different species and age classes. We show a general dominance gradient of body size (larger species to the smaller ones CV > GV > BV > EV), and age (from the adult age class to subadults and juveniles) as the outcome of agonistic encounters between European vultures. Aspects of dominance according to sex and the number of individuals of each species present have not been evaluated. However, according to phenotipic correlates of dominance, in European vultures, as moderate reversed dimorphic species, females could be dominant over males. Complementarily, intraspecific hierarchy seems to be also determined by behavioral factors such as hunger^64,65^.

In summary, the four vulture species studied, and to some extent their age classes, showed differential feeding behaviors that determined the despotic dominance and hierarchies formed during carrion exploitation. The different species selected different types of carrion, depending on the abundance of the available parts. Accordingly, food preferences and availability could also determine the level of agonistic interactions observed.

## Data Availability

Data in which the results of this article are based on is provided within the Dryad Digital Repository (https://datadryad.org/review?doi=doi:10.5061/dryad.52m3q03).
